# Supplementary material for: Simultaneous TCR and IL-2 agonism selectively enhances epitope-specific CD8 T-cell responses during chronic viral infection
Source: J Virol. 2026 Jun 15;100(7):e00593-26. doi: 10.1128/jvi.00593-26 (PMC13366764; doi:10.1128/jvi.00593-26)
Supplement: Supplemental material — Fig. S1 to S3. [file jvi.00593-26-s0001.pdf]

Supplemental Material for

Simultaneous TCR and IL-2 agonism selectively enhances epitope-specific CD8 T-cell responses during chronic viral infection

Masao Hashimoto, Mohammad Affan Khan, Akil Akhtar, Javed N. Agrewala, Gordon J. Freeman, Natasha Girgis, Yu Zhang, Simon Low, Steven N. Quayle, Anish Suri, and Rafi Ahmed

Corresponding author: Rafi Ahmed

Email: rahmed@emory.edu.

This PDF file includes Figures S1-S3.

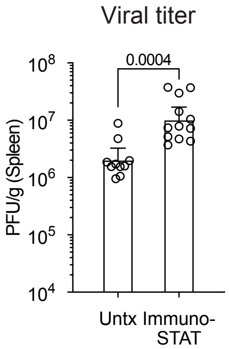

Supplemental Figure 1. Immuno-STAT monotherapy does not improve viral control during chronic LCMV infection. LCMV chronically infected mice (> day 40 post-infection) were left untreated or treated with Immuno-STAT for 7-10 days, followed by analysis of viral titers in the spleen. Results werepooled from 3 experiments with 3-4 mice per group in each experiment. Statistical comparisons were performed using an unpaired Mann-Whitney test. Bars and error bars represent the geometric mean and 95% confidence interval. Untx, untreated.

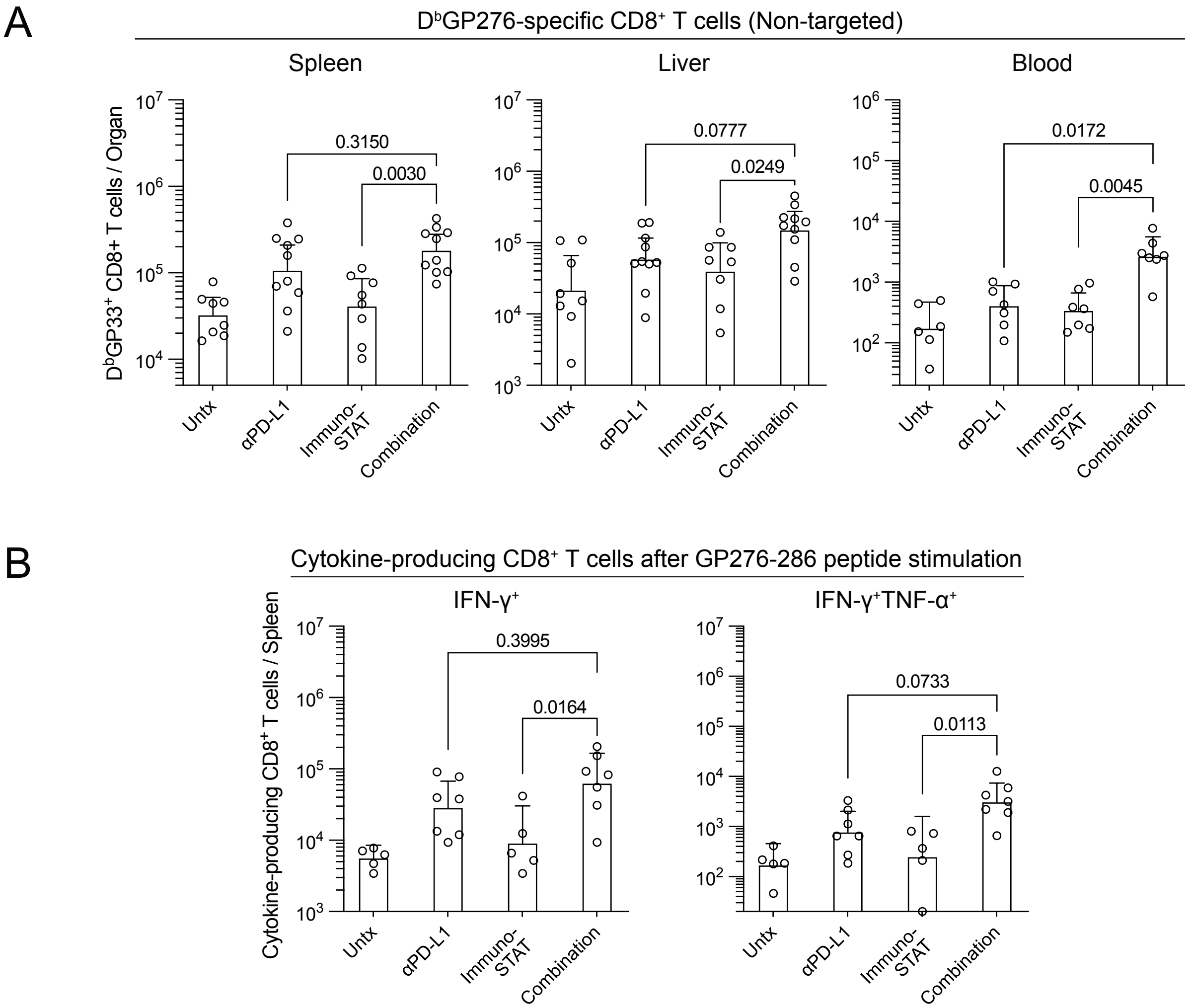

Supplemental Figure 2. Effects of Immuno-STAT alone or in combination with anti-PD-L1 on non-targeted D^bGP276-specific CD8 T cells during chronic LCMV infection.

LCMV chronically infected mice (> day 40 post-infection) were left untreated or treated with anti-PD-L1 antibody alone, Immuno-STAT alone, or a combination of anti-PD-L1 antibody and Immuno-STAT for 10 days, followed by analysis of D^bGP276-specific CD8 T cells. (A) Summary plots showing the number of D^bGP276-specific CD8 T cells in the indicated tissues. (B) Splenocytes from each treatment group were stimulated with GP276-286 peptide for 5 hours, followed by analysis of cytokine-producing CD8 T cells. Results were pooled from 2-3 experiments with n=1-4 mice per group in each experiment. Statistical comparisons were performed using the Kruskal-Wallis test with Dunn's correction (A, B). Bars and error bars represent the geometric mean and 95% confidence interval (A, B).

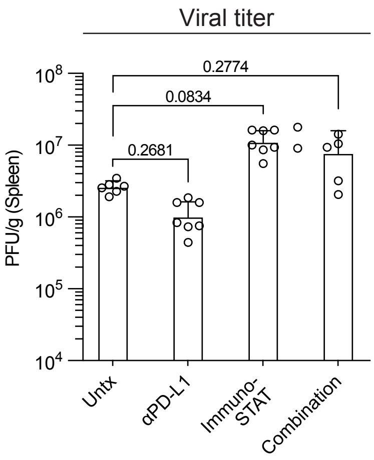

Supplemental Figure 3. Immuno-STAT alone or in combination with anti-PD-L1 does not improve viral control in a stringent chronic LCMV model.
Chronically LCMV-infected mice (> 40 days post-infection) were left untreated or treated with anti-PD-L1 antibody alone, Immuno-STAT alone, or a combination of anti-PD-L1 antibody and Immuno-STAT for 10 days, followed by analysis of viral titers in the spleen. Results were pooled from 2 experiments with 3-4 mice per group in each experiment. Statistical comparisons were performed using the Kruskal-Wallis test with Dunn's correction for multiple comparisons. Bars and error bars represent the geometric mean and 95% confidence interval. Untx, untreated.
